# Editorial: Genetically mobile elements repurposed by nature and biotechnologists

**DOI:** 10.3389/fmolb.2022.992664

**Published:** 2022-08-29

**Authors:** Christopher W. Lennon, Brian P. Callahan, Benoit Cousineau, David R. Edgell, Marlene Belfort

**Affiliations:** ^1^ Department of Biological Sciences, Murray State University, Murray, KY, United States; ^2^ Department of Chemistry, Binghamton University, State University of New York, Binghamton, NY, United States; ^3^ Department of Microbiology and Immunology, McGill University, Montréal, QC, Canada; ^4^ Department of Biochemistry, Schulich School of Medicine and Dentistry, The University of Western Ontario, London, ON, Canada; ^5^ Department of Biological Sciences and RNA Institute, University at Albany, Albany, NY, United States

**Keywords:** mobile genetic elements, introns, inteins, CRISPR, biotechnology

**FIGURE 1:**
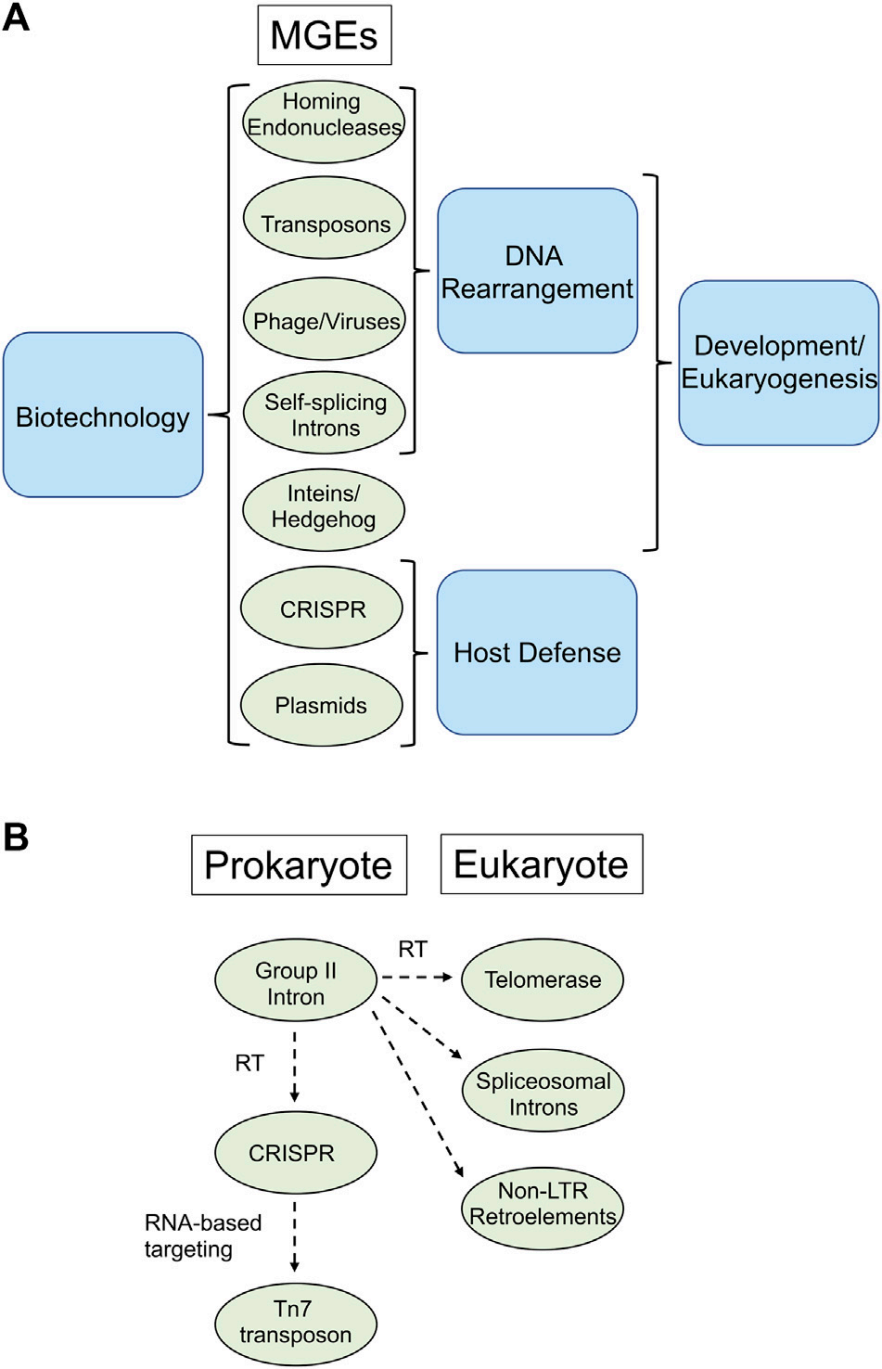
MGE’s repurposed by nature and biotechnologists. **(A)**. Processes mediated by MGE’s. All the MGE’s listed are used in biotechnological application (left), whereas various groups of MGE’s are involved in DNA rearrangements, host defense, eukaryogenesis and development (right). **(B)**. Gene flow among MGE’s. An example of exaptation is provided by group II introns, which harbor a reverse transcriptase (RT) that is closely related to the RT of telomerase in eukaryotes, spliceosomal introns and non-LTR retroelements. Likewise, CRISPR-associated RTs are likely derived from group II introns, whereas some Tn7 transposons have inherited RNA-guided targeting from CRISPR elements.

Mobile genetic elements (MGEs), including introns, retroelements, and inteins are present in many microbes. These elements, traditionally considered molecular parasites, reshape genomes and drive evolution. However, non-random distributions within host genomes and response to environmental cues force the rethinking of MGEs as simple parasites, raising questions about the possible importance of these elements to the host organisms they invade (Belfort 2017). Indeed, compelling evidence indicates that nature has repurposed these MGEs with spectacular results, leading to such evolutionary marvels as CRISPR immune systems to protect bacteria from phage, spliceosomal introns and hedgehog signaling. These mobile elements have also proven exceptionally useful to biotechnologists, leading to the development of ground-breaking technologies in genome manipulation, next-generation sequencing, protein engineering, and synthetic biology (Enyeart et al., 2014; Stoddard 2014; Sarmiento and Camarero 2019). This Research Topic, which includes one opinion, three original research, and five review articles, provides a contemporary perspective on the numerous areas of interest regarding the biological importance of MGEs to the hosts they invade and also highlights new and exciting applications of these elements by biotechnologists (Figure 1A).

In their review, Benler and Koonin offer a first comprehensive picture of cellular functions that may be evolutionarily derived from repurposed autonomous MGEs, a process called exaptation. Organisms tend not to purge MGEs but often exapt them, to selective advantage. Cellular roles to which MGEs contribute is broad, with common functionalities often mediating different types of biological conflicts, as for example CRISPR systems that furnish adaptive immunity in bacteria. Conversely, some Tn7 family transposons have inherited CRISPR RNA-guided mechanisms for target choice. There is also a CRISPR-associated reverse transcriptase (RT) that is most closely related to the RT of self-splicing group II introns, providing another example of inter-MGE exaptation. Likewise, telomerase, which restores chromosomal termini and the key protein of the eukaryotic spliceosome were captured from an RT encoded by group II introns, which are bacterial retrotransposons (Figure 1B). Indeed, there appears to be a constant, bidirectional flow of genes between MGEs and among MGEs and their host genomes.

The abovementioned group II introns and retrotransposons are covered in three papers. In the first, mechanistic work by Molina-Sanchez et al. shows that the RmInt1 intron from *Sinorhizobium meliloti* preferentially binds the template strand during lagging strand DNA synthesis non-specifically and that the binding sites are preferentially positioned around the origin of replication. They hypothesize that the positioning on the genome may be influenced by intron distribution in the cell during DNA replication, interactions of the intron with components of the DNA replication machinery, and chromatin accessibility. Overall, this work reinforces the link between replication and group II intron mobility and should be considered when engineering efficient group II intron “targetrons” for high fidelity chromosome insertion.

In a second paper, Costa discusses two examples of the structural versatility of group II introns based on recent 3D structures (Toor et al., 2008; Qu et al., 2016) highlighting the remarkable flexibility of these large, structured ribozymes during splicing and mobility. She first focuses on the plasticity of the 3′ end of the intron, suggesting that this structural flexibility may be the origin of a potential alternative splicing system in bacteria. Second, the relevance of a novel base-pairing interaction between the intron RNA and the DNA target site during retrohoming is discussed, adding another layer of mechanistic nuance to engineering group II intron targetrons.

Third, chromosomal retrotransposons, like group II introns and yeast Ty retrovirus-like elements, must be silenced to prevent promiscuous genomic movement while also enabling mobility in response to cellular stresses. Different genetic screens have identified large, mostly non-overlapping sets of host factors that modulate Ty1 mobilization. A paper by Salinero et al. defines a distinct classes of host factors that act under specific conditions, accounting for the lack of overlap among Ty1 host factors, and demonstrate that Ty1 promoter-dependent factors can affect not only the level but also the fate of Ty1 RNA.

Five papers focus on inteins (intervening proteins) and the related eukaryotic Hedgehog (Hh). Inteins are MGEs due to the activities of nested site-specific endonucleases. Beyer and Iwai detail how structural diversity of intein accessory domains can influence DNA cleavage by these endonucleases. This study has implications for the engineering of endonucleases with new and custom cleavage specificity, as well as for the evolutionary persistence of mobile inteins within organisms.

As MGE’s, inteins are often housed within essential proteins, whereby inhibition of splicing would compromise the ability of the intein-containing host to survive (Novikova et al., 2016). As inteins are absent in humans and widespread in microbes, these elements represent attractive drug targets. Several human pathogens, including *Mycobacterium tuberculosis* and *Cryptococcus neoformans* that kill over 1.5 million people annually contain inteins within essential genes. The paper by Wall et al. reviews established screening methods to monitor protein splicing in the presence of potential inhibitors. Additionally, they address possible off-target events in the development of novel drugs to target intein-containing pathogens due to the structural and mechanistic similarity of inteins to the Hh signaling protein, a protein critical in human development and required for proper cell differentiation. Tharappel et al. provide a detailed review of inteins in human pathogens and inteins as well as Hh inhibitors. Next, they discuss the utility of inteins in therapeutics and drug discovery. Together, these two articles provide an in-depth examination of screening methods, novel antimicrobials, and inteins as tools to aide in drug discovery.

Inteins are also important tools in biotechnology. Prabhala et al. provide a concise historical overview of the intellectual property landscape encompassing the application of inteins as “self-cleaving affinity tags” for the preparation of native proteins. This insider’s perspective starts with intein-based purification approaches patented in 1996 by New England Biolabs, up through recent patents to academic and to industry labs where inteins are applied for installing chemical modifications and for the scaled-up isolation of homogeneous therapeutic proteins.

Precursor forms of eukaryotic Hh proteins appear to owe their specialized self-cleaving/protein-cholesterol splicing activity to a domesticated MGE-like the **h**edgehog/**int**ein (HINT) domain. Compared to intein splicing, our understanding of the hedgehog reaction is woefully behind in several areas: structure/function, chemical probes, and biotechnology applications (Ingham 2022). Kandel and Wang bring us up-to-date on the latest results while laying bare the many gaps that exist.

This Research Topic covers exciting recent advances in the MGE field, including their evolutionary repurposing, Additionally, the compilation considers self-splicing introns and inteins, MGE’s that possess the unique ability to perform chemistries that have proved invaluable to numerous biotechnological applications, and that also serve as promising therapeutic targets.
